# Antisynthetase Syndrome With Cardiac Involvement: Role of Cardiac Magnetic Resonance Imaging in Its Diagnosis and Management

**DOI:** 10.7759/cureus.80904

**Published:** 2025-03-20

**Authors:** Jonathan Lopez, Ralph Matar

**Affiliations:** 1 Cardiology, University of Florida College of Medicine, Gainesville, USA

**Keywords:** autoimmune, cardio, cardiovascular anomalies, inflammatory cardiomyopathy, mri cardiac, non-ischemic cardio

## Abstract

We present a case of newly diagnosed heart failure with reduced ejection fraction secondary to antisynthetase syndrome (ATS) in a 63-year-old female patient. The patient presented with symptoms and laboratory findings indicative of an ATS flare, which included inflammatory arthritis, muscular involvement, and exertional dyspnea. A systematic and structured diagnostic approach was undertaken, commencing with transthoracic echocardiography (TTE) to evaluate cardiac function and morphology. This was followed by ischemic assessment through coronary angiography to rule out ischemic heart disease, and subsequently, cardiac magnetic resonance imaging (CMR) based on findings from the TTE.

The initial TTE revealed a newly reduced ejection fraction of 30-35%, a significant decline from previous normal echocardiograms. The ischemic evaluation identified mild, non-obstructive coronary artery disease, effectively excluding ischemic cardiomyopathy as a contributing factor. CMR was pursued due to concerns for myocarditis and provided further diagnostic clarity, revealing impaired left ventricular (LV) systolic function and near-transmural late gadolinium enhancement (LGE) in the inferolateral and anterolateral wall segments, along with subendocardial LGE in the inferoseptal wall segment. These findings were consistent with regional wall motion abnormalities observed on TTE. In response, the patient's baseline immunosuppressive therapy was optimized with the addition of mycophenolic acid. No additional etiologies were identified to account for the new LV dysfunction.

Following optimization of immunosuppressive therapy and heart failure management, the patient exhibited significant clinical improvement, including stabilization of cardiac function and resolution of inflammatory markers. This case report adds to sparse literature describing ATS-induced myocarditis and helps highlight integration of advanced imaging modalities like CMR, which provides critical insights into myocardial tissue characterization and helps guide management strategies.

## Introduction

Antisynthetase syndrome (ATS) is a rare autoimmune myopathy characterized by the presence of autoantibodies targeting tRNA synthetases. Its clinical presentation typically includes interstitial lung disease, myositis, arthritis, and Raynaud’s phenomenon [1]. Although cardiac involvement, such as myocarditis, has been described in small cohorts, its prevalence ranges from 3 to 30%, depending on diagnostic criteria and study populations [2,3].

Patients presenting with newly identified left ventricular (LV) dysfunction require urgent evaluation to ensure timely and effective management. ATS-induced myocarditis should remain a key consideration in the differential diagnosis for such patients. Maintaining a high index of suspicion facilitates informed therapeutic decisions, guiding treatment strategies and enabling the prompt initiation of immunosuppressive therapy when warranted.

Cardiac magnetic resonance imaging (CMR) is regarded as the imaging modality of choice in this context. Its superior sensitivity and specificity allow for detailed myocardial tissue characterization, including the detection of late gadolinium enhancement (LGE) and myocardial edema, findings that might be overlooked with alternative modalities such as transthoracic echocardiography (TTE) or nuclear imaging. This advanced imaging capability ensures precise diagnosis and optimal management of ATS-related cardiac complications.

## Case presentation

A 63-year-old woman presented to our outpatient cardiology clinic with a newly diagnosed case of heart failure with reduced ejection fraction (HFrEF) and an ejection fraction (EF) of 30-35%. Her diagnosis of ATS had been established approximately two years prior, during which she exhibited inflammatory arthritis, bilateral proximal muscle weakness, and exertional dyspnea. Laboratory findings at the time revealed elevated creatine kinase (CK) and aldolase levels, alongside positive anti-Jo1 and antinuclear antibodies. High-resolution computed tomography demonstrated findings consistent with nonspecific interstitial pneumonia. She responded well to her initial immunosuppressive regimen (initiated immediately after ATS diagnosis), which included glucocorticoids and rituximab, resulting in significant clinical improvement.

The patient remained in remission until six months prior to our evaluation when she experienced an ATS flare. At this time, she reported worsening polyarthralgia, diffuse weakness, myalgias, and increased difficulty with activities such as climbing stairs. She also developed worsening shortness of breath with minimal exertion. Laboratory investigations revealed an elevated CK level of 976 U/L (normal range 24-173 U/L). In response, her Rheumatology team intensified her immunosuppressive therapy with the addition of mycophenolic acid. A TTE performed during this period revealed a newly reduced EF of 30-35%, diastolic dysfunction, and hypokinesis of the inferolateral and anterolateral wall segments, in contrast to her previously normal TTE findings. She was subsequently referred to cardiology for further assessment and management.

During her cardiology evaluation, the patient expressed significant difficulty performing her usual activities. At baseline, she led an active lifestyle, including horseback riding, weightlifting, and walking without any symptoms of exertional chest pain or discomfort. However, she now experienced pronounced exertional shortness of breath with minimal activities, such as walking within her home. She reported mild intermittent lower extremity swelling but denied orthopnea, paroxysmal nocturnal dyspnea, chest pain, diaphoresis, syncope, or recent infectious or viral symptoms. Her social history was unremarkable, as she had never smoked and consumed alcohol only on rare social occasions. On physical examination, she appeared euvolemic.

To exclude ischemic cardiomyopathy as the cause of her reduced EF, a coronary angiogram was performed. A non-invasive ischemic workup, such as a stress test, was not pursued in this case due to the patient's clinical presentation, which warranted a more definitive approach to rule out ischemic heart disease. Additionally, she was initiated on a low-dose beta blocker and an angiotensin-converting enzyme (ACE) inhibitor. Further investigations were carried out to guide management and refine her diagnosis.

Investigations 

ECG revealed sinus rhythm with a ventricular rate of 77. There was no evidence of prolonged PR segments or QRS/QT intervals. There was no evidence of acute ischemic changes or Q waves to suggest current or prior myocardial infarction. There was absence of left ventricular hypertrophy.

TTE as mentioned prior revealed new systolic HF with anterolateral and inferolateral hypokinesis. There was no evidence of RV dysfunction. Tricuspid annular plane systolic excursion was 1.97 cm.

Troponins were within normal limits and consistently <6 PG/ML (reference range: <15 PG/ML). Hemoglobin was within normal limits at 12.1 G/DL (reference range 12.0 - 16.0 G/DL). Thyroid-stimulating hormone (TSH) was within normal limits at 1.638 MIU/L (reference range: 0.400 - 5.000 MIU/L). B-Natriuretic peptide was elevated at 166 PG/ML (reference range:<100 PG/ML).

A coronary angiogram revealed only mild nonobstructive CAD. She was continued on guideline-directed medical therapy (GDMT) and an immunosuppression regimen. 

**Figure 1:**
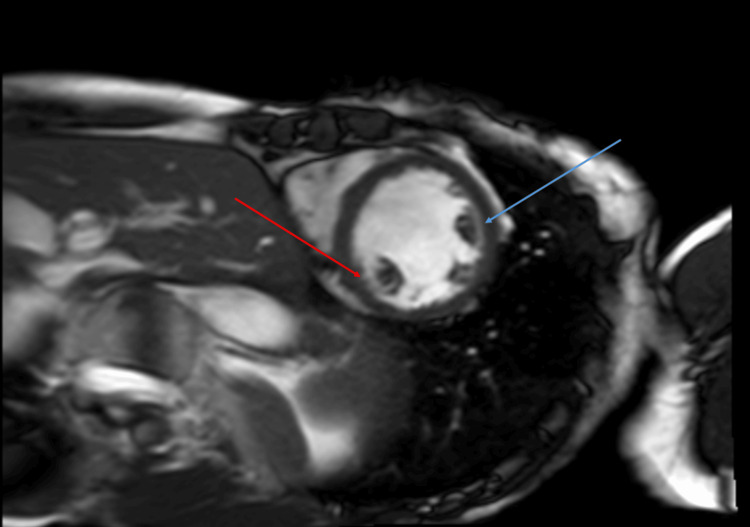
Cardiac MRI short axis: Phase-sensitive inversion recovery (PSIR) Red arrow: Subendocardial late gadolinium enhancement involving the mid-inferoseptal wall segment. Blue arrow: Mid-myocardial late gadolinium enhancement involving the mid-anterolateral wall segment.

**Figure 2:**
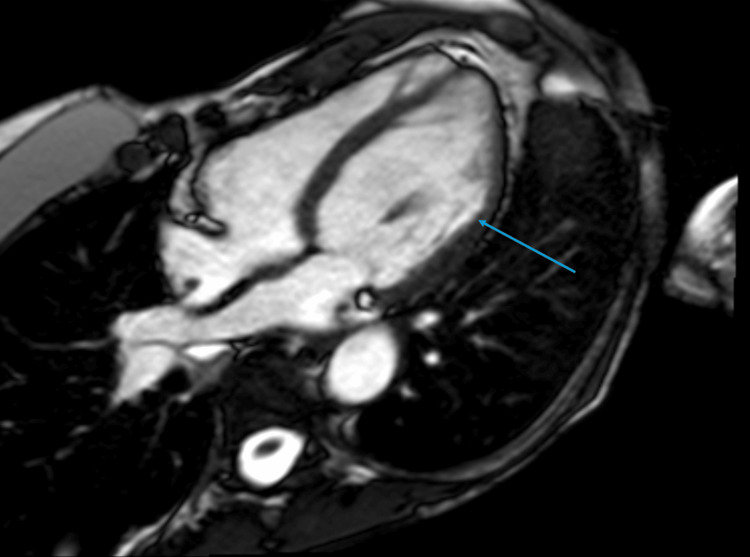
Cardiac MRI: apical four-chamber PSIR Blue arrow: Mid-myocardial, near transmural late gadolinium enhancement involving the mid-anterolateral and apical lateral wall segments. PSIR: Phase-sensitive inversion recovery

CMR (Figure 1 and Figure 2) was performed which showed depressed LV systolic function (EF 41%) with near transmural mid-myocardial LGE in the inferolateral and anterolateral wall segments in addition to subendocardial LGE in the inferoseptal wall. This correlated with previous areas of regional wall motion abnormalities seen on TTE. There was no hyperintense signal on T2-weighted imaging to suggest ongoing edema. CMR findings were suggestive of replacement fibrosis likely secondary to myocarditis in the absence of active inflammation. The extent of fibrosis was thought to correlate with her clinical severity.

Differential diagnosis

A 63-year-old woman presenting with newly diagnosed cardiomyopathy and reduced LV systolic function necessitates a comprehensive differential diagnosis. In the absence of obstructive coronary artery disease (CAD) and valvular heart disease, potential etiologies to consider include stress-induced cardiomyopathy (“Takotsubo”), toxicity-induced cardiomyopathy, infiltrative or genetic cardiomyopathies, high-output cardiomyopathy, or cardiac involvement secondary to systemic inflammatory disease.

In this case, there was no history of an emotional or physical stressor, and the patient’s subacute presentation contrasted with the more acute and rapidly progressive symptoms typical of stress-induced cardiomyopathy. The hallmark symptoms of Takotsubo cardiomyopathy (chest pain, dyspnea, and syncope) resulting from acute catecholamine surge and myocardial stunning, were absent [4]. Furthermore, the characteristic regional wall motion abnormalities associated with stress-induced cardiomyopathy were not observed on the patient’s TTE. The patient did not meet the Mayo modified diagnostic criteria for Takotsubo cardiomyopathy [5].

Infiltrative diseases, such as amyloidosis and sarcoidosis, as well as genetic cardiomyopathies like hypertrophic and dilated cardiomyopathies, were deemed less likely due to the lack of characteristic findings on multiple imaging modalities. High-output cardiomyopathy was excluded based on normal TSH and hemoglobin levels, ruling out hyperthyroidism and anemia as potential contributors. There were no findings of arteriovenous shunts, tachycardia, or arrhythmias throughout the evaluation to support this diagnosis. Viral myocarditis was considered as a potential etiology; however, the absence of active or recent viral infections and the lack of a preceding viral prodrome in the patient’s clinical history made this diagnosis less likely. COVID-19 nucleic acid amplification tests were negative, and given the low clinical suspicion, further detailed serologic viral testing was not pursued.

Toxicity-induced cardiomyopathy related to the patient’s rituximab regimen was considered but ultimately deemed unlikely. The patient had tolerated multiple cycles of rituximab without previous cardiovascular complications and experienced clinical improvement in her ATS symptoms with this therapy. Reported adverse cardiovascular effects of rituximab, including angina, acute coronary syndrome, and various arrhythmias (e.g., monomorphic ventricular tachycardia, supraventricular tachycardia, bradycardia, atrial fibrillation), typically occur during the initial infusions or within the first few months of therapy [6]. The absence of these adverse effects over the course of treatment made rituximab-induced cardiomyopathy less probable.

Treatment 

Mycophenolic acid was selected for managing the ATS flare due to its efficacy in controlling autoimmune inflammation while offering a favorable side effect profile compared to other immunosuppressive agents. It is particularly effective in reducing immune hyperactivity and maintaining remission in autoimmune conditions, making it a preferred choice over alternatives like cyclophosphamide, which carries a higher risk of toxicity. Mycophenolic acid was up-titrated over the course of weeks to 1 gram twice a day. This was in addition to her active regimen of prednisone (at increased dosage) and the maintenance rituximab infusions she was previously receiving. 

When seen by our clinic, she was started on GDMT with metoprolol and lisinopril. A switch from lisinopril to valsartan was driven by the patient's development of a persistent dry cough, a well-documented side effect of ACE inhibitors like lisinopril. The cough significantly impacted the patient’s adherence to the medication regimen, as it interfered with overall quality of life. Valsartan, an angiotensin II receptor blocker (ARB), was chosen as an alternative due to its similar efficacy in managing heart failure and hypertension, with a lower risk of inducing cough. Beta blockers and ARBs were initiated at low doses and gradually uptitrated over a period of 3-6 months between her cardiology visits. 

Follow-up imaging and clinical evaluations were planned to monitor myocardial recovery. A repeat TTE was scheduled three months post-intervention to assess LV function.

Outcome and follow-up 

Over the course of two years, the patient remained under the care of Rheumatology and our Cardiology clinic, demonstrating steady improvement in her myalgias, arthralgias, breathing, and heart failure symptoms. Her functional status significantly improved, and her most recent TTE revealed partial recovery of her EF now at 45-50%.

Follow-up cardiac MRI performed about two years following ATS myocarditis diagnosis showed no significant changes in the extent or distribution of LGE, indicating persistent fibrosis. This underscores the prognostic significance of myocardial fibrosis, which remains a strong predictor of limited reversibility of ventricular dysfunction. Of note, by the time of her follow-up CMR, the patient’s CK levels had normalized completely, further indicating effective disease control.

The partial recovery of EF despite adherence to GDMT could be attributed to several factors including persistent myocardial fibrosis, identified by LGE on follow-up CMR.

## Discussion

Myocarditis, characterized by inflammatory infiltration of the myocardium, encompasses a wide spectrum of infectious etiologies (including viral, bacterial, fungal, and protozoal causes) and noninfectious triggers (such as cardiotoxins, hypersensitivity reactions, and systemic inflammatory disorders). Its clinical significance lies in its capacity to induce inflammatory cardiomyopathy, leading to severe LV systolic dysfunction, heart failure, arrhythmias, and cardiogenic shock, which can manifest as a fatal presentation [7].

In this case, after ruling out alternative etiologies, the patient’s acute exacerbation of ATS correlated with the decline in EF, supporting a diagnosis of ATS-associated myocarditis. This conclusion was further reinforced by the patient's improvement in symptoms and LV function following the escalation of immunosuppressive therapy, as well as the presence of regional wall motion abnormalities and patterns of LGE including replacement fibrosis visualized on CMR. These findings were observed in the absence of epicardial CAD. While endomyocardial biopsy (EMB) remains the gold standard for diagnosing myocarditis, CMR has become an invaluable tool for noninvasive diagnosis in this case. The Lake Louise Criteria serve as the foundation for diagnosing myocarditis via CMR with our patient possessing LGE and T1-based imaging patterns, consistent with myocardial damage. Given the clinical and imaging data supporting the diagnosis, EMB was not pursued due to the invasive nature of the procedure and the patient's favorable response to pharmacological management.

This case underscores the importance of maintaining a broad differential diagnosis when evaluating patients with cardiomyopathy and newly reduced LV systolic function. Autoimmune myocarditis, particularly in the context of systemic inflammatory diseases like ATS, should always be considered, even in light of its relatively lower reported prevalence. Recognizing myocardial involvement in ATS can inform treatment regimens, enabling timely initiation of immunosuppression and heart failure therapies to prevent irreversible myocardial damage. Follow-up CMR in this case confirmed the absence of active myocardial edema or inflammation (with no hyperintense T2-weighted signal), validating the effectiveness of our therapeutic approach. This success was achieved primarily through the adjustment of immunosuppressive therapy, complemented by GDMT therapies. 

A review of cases has estimated the prevalence of myocarditis in ATS patients to range from 3 to 30%. Proposed mechanisms include autoimmune-mediated direct myocyte injury and vascular inflammation, reflecting the complex interplay between autoimmunity and cardiac pathology [2,3,8]. A registry involving 352 ATS patients highlighted that cardiac involvement, though underreported, may be more prevalent than previously recognized [3]. Case reports describe varied presentations, ranging from severe biventricular heart failure progressing to cardiogenic shock and cardiac arrest in some patients to more subacute manifestations like dyspnea, abnormal ECG/troponin levels, and LV dysfunction. Importantly, these subacute presentations often led to a new diagnosis of ATS, followed by immunosuppression and subsequent symptom improvement [9].

Such findings reinforce the necessity of considering ATS-related myocarditis not only in patients with established ATS diagnoses but also in those presenting with unexplained LV dysfunction and systemic inflammatory features. Timely recognition and aggressive treatment are crucial, as myocarditis can lead to irreversible structural damage and long-term cardiac complications. By maintaining a heightened level of suspicion, clinicians can identify patients who may benefit from intensified immunosuppression and GDMT, offering them the best opportunity for myocardial recovery and improved outcomes.

## Conclusions

In cases of LV systolic dysfunction and HFrEF symptoms in a middle-aged woman with a history of autoimmune disease, a comprehensive differential diagnosis is essential. This should include consideration of immune-mediated myocarditis. While EMB remains the gold standard for diagnosis of myocarditis, CMR is the non-invasive imaging modality of choice and holds both diagnostic and prognostic significance. For example, the presence and extent of LGE may correlate with long-term outcomes, including the likelihood of ejection fraction EF recovery, ventricular remodeling, and the risk of adverse cardiac events. Persistent fibrosis on follow-up CMR may also signal limited functional recovery despite therapy, emphasizing the importance of early intervention. Long-term monitoring and follow-up including repeat CMR to assess for resolution of active inflammation, reduction in edema, and progression or stabilization of fibrosis as well as periodic TTE have not been standardized in ATS myocarditis patients. 

A multidisciplinary approach is paramount in these complex cases. Cardiologists play a central role in leading the cardiovascular workup, optimizing GDMT, and monitoring cardiac function. Rheumatologists provide expertise in managing autoimmune pathology and tailoring immunosuppressive regimens based on the patient’s systemic disease activity. By emphasizing a tailored, coordinated approach and leveraging the diagnostic and prognostic capabilities of CMR, clinicians can ensure optimal management of ATS-associated myocarditis while also paving the way for improved care in other autoimmune conditions with cardiac involvement. This highlights the need for vigilance, collaboration, and timely intervention in cases of unexplained cardiomyopathy with a suspected immune-mediated component.
